# Targeting mitochondrial quality control to disarm phytopathogenic fungi

**DOI:** 10.1080/21505594.2026.2757956

**Published:** 2026-11-09

**Authors:** Horchelle T. Djomptchouang, Zhong Zhang, Yang Bi, Dov Prusky, Patrick B. Njobeh, Thierry Y. Fonkui, Hui Wang

**Affiliations:** a College of Food Science and Engineering, Gansu Agricultural University, Lanzhou, China; b Department of Postharvest Science of Fresh Produce, Agricultural Research Organization-The 12 Volcani Center, Rishon LeZion, Israel; c Department of Biotechnology and Food Technology, Faculty of Science, University of Johannesburg, Johannesburg, South Africa

**Keywords:** Phytopathogenic fungi, mitochondrial quality control, mitophagy, mitochondrial dynamics, fungal pathogenicity, antifungal targets

## Abstract

Fungal diseases cause substantial crop losses worldwide, while increasing fungicide resistance highlights the need for new disease-control strategies. Mitochondrial quality control (MQC), comprising mitochondrial dynamics, biogenesis, proteostasis, and mitophagy, maintains mitochondrial integrity and functional capacity during infection. Evidence from phytopathogenic fungi links disruption of individual MQC mechanisms to defects in growth, stress adaptation, and pathogenicity. Here, we synthesize evidence across hemibiotrophic, vascular, and necrotrophic pathogens and across distinct stages of the infection cycle. Because mechanistic evidence is strongest in *Magnaporthe oryzae*, we use it as a principal reference system while highlighting conserved and lifestyle-specific requirements emerging from other fungi. This review proposes three complementary strategies for exploiting MQC vulnerabilities: targeting pathogen-specific regulatory features within conserved mitochondrial systems, exploiting interdependencies among MQC processes and mitochondrial stress responses, and targeting stage-specific mitochondrial requirements as potential intervention points. We also highlight some limitations that need resolution while considering MQC mechanisms as antifungal targets.

## Graphical abstract

**Figure:**
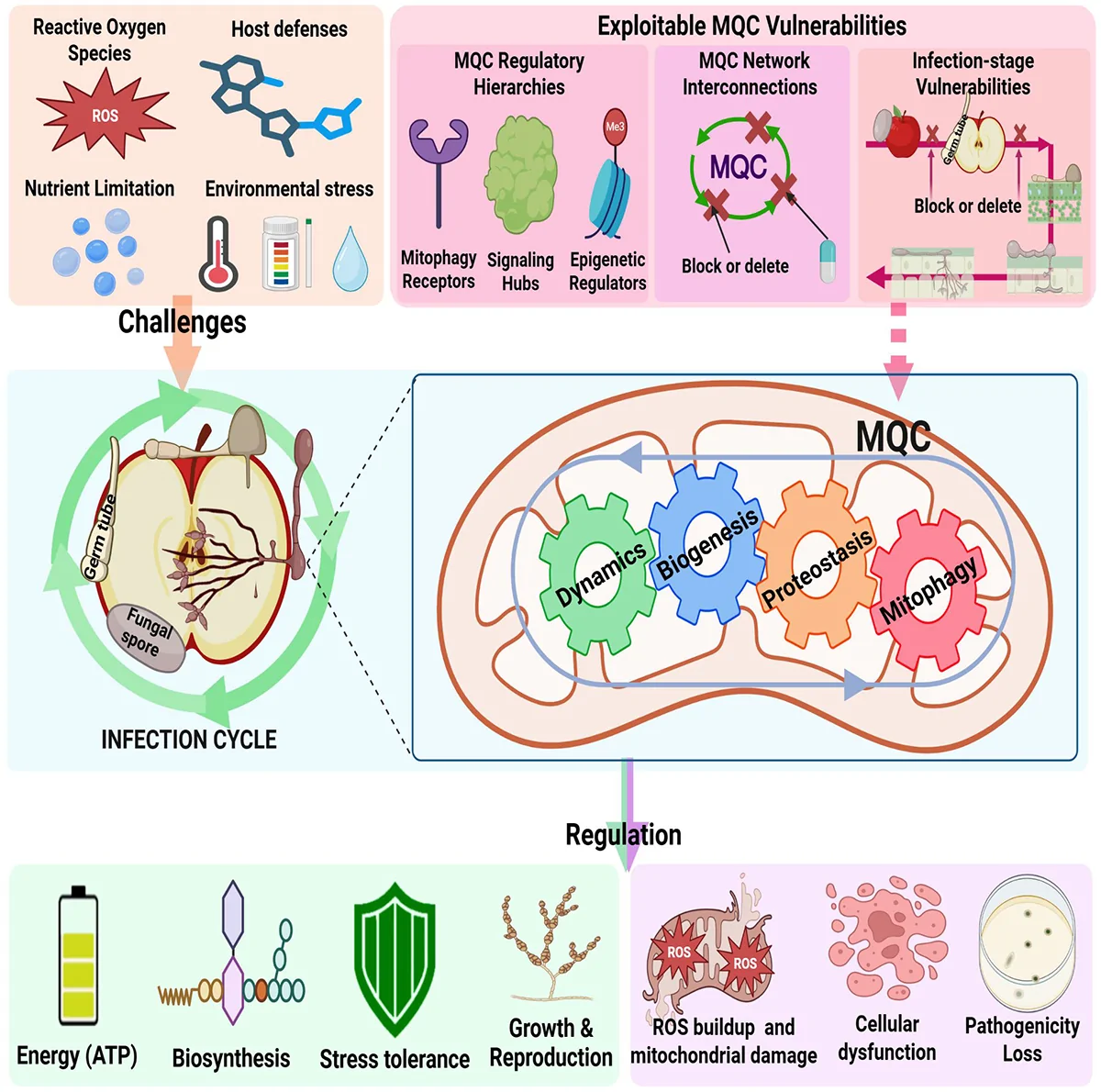

## Introduction

Phytopathogenic fungi pose a critical threat to global agriculture and food security, accounting for annual yield losses of 10–20% in major crops and inflicting economic damages amounting to tens of billions of US dollars [1,2]. Current disease management relies heavily on chemical fungicides, leading to increasing resistance to multiple fungicide classes in key pathogens: the emergence of azole-resistant *Aspergillus fumigatus* strains in agricultural settings directly links fungicide use to a public health threat [3]. Moreover, resistance in the rice blast fungus *Magnaporthe oryzae* continues to escalate; eight distinct point mutations in succinate dehydrogenase genes recently identified confer high resistance to the novel succinate dehydrogenase inhibitor-based fungicide pydiflumetofen, with resistance factors exceeding 213 [4]; therefore, safer, sustainable antifungals are urgently needed. Targeting fundamental biological processes essential to fungal pathogenicity is a promising avenue, and mitochondria have emerged as a compelling target in this regard [5–7].

Far more than simple powerhouses, mitochondria serve as indispensable hubs for energy production, biosynthetic precursor generation, redox homeostasis, and signaling; functions that are critically amplified during the intense metabolic demands of host invasion [8–10]. This central role, however, creates an inherent vulnerability: mitochondria are both sources and targets of reactive oxygen species (ROS). Their heightened activity during infection generates endogenous ROS; on the other hand, plant defense responses, including oxidative bursts and phytoalexins, impose exogenous oxidative stress, together threatening mitochondrial integrity, which in turn could trigger cell death [11]. The success of a fungal pathogen therefore depends, to varying degrees, on its ability to maintain functional mitochondria under this continuous assault. To do so, fungi employ an integrated network of surveillance and repair pathways collectively known as mitochondrial quality control (MQC). This system encompasses mitochondrial dynamics (fusion and fission), biogenesis, proteostasis, and mitophagy, and works to regulate organelle number, remove damaged components, and adapt the network to metabolic demands [12–15].

MQC is therefore closely linked to fungal pathogenicity because infection requires mitochondria to remain functionally competent while undergoing repeated developmental transitions and host-imposed stresses [15]. Evidence across phytopathogenic fungi shows that mitochondrial dynamics, biogenesis, proteostasis, and mitophagy contribute to infection-related processes including germination, appressorium development, host penetration, invasive growth, stress adaptation, and reproduction. Although the available evidence is strongest for selected MQC mechanisms and pathogens, complementary studies of mitochondrial respiration, metabolism, transport, and redox homeostasis further highlight the functional capacity that must be maintained during infection. This review integrates these findings across hemibiotrophic, necrotrophic, vascular, and postharvest pathogens to define how MQC supports mitochondrial competence throughout the infection cycle. Building on this biological framework, we examine three ways to exploit MQC dependence for disease control: targeting pathogen-specific regulatory features within conserved MQC machinery, exploiting interconnections among MQC processes and mitochondrial stress responses, and targeting stage-specific mitochondrial requirements during infection. We further consider the progression from genetic evidence to chemical target engagement, fungal–host selectivity, and in planta efficacy, thereby linking the mechanistic importance of MQC to its potential as a source of new antifungal strategies.

### Core functions of the mitochondrion in fungal pathogenesis

Mitochondria play a central role in fungal pathogenicity; beyond their canonical role in ATP synthesis, they coordinate biosynthetic pathways, intracellular signaling, and cell-fate decisions. During infection, however, mitochondria also face substantial metabolic and host-derived stresses that can compromise their integrity and function. This combination of essentiality and vulnerability makes mitochondrial processes relevant to understanding fungal pathogenicity and provides a biological basis for investigating mitochondria-associated processes as potential antifungal targets.

### Structural and functional plasticity: a network adapted for invasion

Mitochondria are dynamic organelles with a double-membrane architecture that creates specialized compartments: the outer membrane functions as a platform for molecular exchange and signaling, whereas the inner membrane folds into cristae that house the electron transport chain (ETC) and ATP synthase [8,16]. In phytopathogenic fungi, the inner mitochondrial membrane also supports a flexible respiratory system that can include alternative oxidase (AOX) and multiple NADH dehydrogenases, providing metabolic versatility and contributing to respiratory and redox adaptation under host-imposed stress. This can reduce ETC over-reduction and limit respiratory ROS accumulation while maintaining metabolic activity under stress, therefore contributing to infection [17–19]. For instance, AOX contributes to appressoria formation in *M. oryzae* under oxidative stress, linking respiratory flexibility to a key step in fungal infection [20]. Similarly, in the necrotroph *Botrytis cinerea*, deletion of BcAOX caused defects in mycelial growth, sporulation, and spore germination, together with increased sensitivity to oxidative stress and quinone-outside inhibitor fungicides [21]. These findings show how respiratory plasticity can shape fungal development, stress adaptation, and virulence across different pathogenic lifestyles.

Beyond energy production, mitochondria also help regulate fungal cell fate. The mitochondrial intermembrane space contains cytochrome c, whose redistribution to the cytosol under mitochondrial stress can contribute to programmed cell death (PCD) pathways, linking mitochondrial integrity to cell survival [22]. The mitochondrial matrix contains the mitochondrial genome, which encodes a subset of proteins required for oxidative phosphorylation. Consequently, mitochondrial genome integrity and variation can influence respiratory function and, traits relevant to fungal fitness, including virulence and antifungal resistance [23–25]. Studies of the inner membrane protein FgLetm1 in *Fusarium graminearum* further underscore the importance of mitochondrial structural integrity for pathogenesis. Deletion of FgLetm1 resulted in vegetative growth defects, reduced endogenous ROS production, decreased deoxynivalenol mycotoxin biosynthesis, and attenuated virulence in wheat [26].

### A multifunctional hub: metabolism, signaling, and cell fate

In eukaryotes, mitochondria contribute not only to ATP production through the Electron Transport Chain (ETC), but also to a range of catabolic and biosynthetic processes. The tricarboxylic acid cycle (TCA) generates reducing agents to support oxidative phosphorylation and provides carbon intermediates for the biosynthesis of amino acids, lipids, nucleotides, and other cellular metabolites. These metabolic outputs support the energetic and biosynthetic demands of infection and contribute to processes such as stress adaptation, cell wall remodeling, and host colonization [8,27].

Mitochondrial metabolism also generates reactive oxygen species (ROS), including superoxide and hydrogen peroxide. At controlled levels, those compounds can act as signaling molecules, but excessive accumulation under metabolic or environmental stress can damage proteins, lipids, membranes, and nucleic acids, contributing to cellular dysfunction or death. Efficient mitochondrial metabolism and appropriate redox regulation are therefore important for fungal survival during developmental and infection-associated transitions. Conversely, persistent damage to mitochondrial DNA or the respiratory machinery components can compromise mitochondrial function and reduce fungal fitness and pathogenic potential [7,28].

While essential for fungal survival, compromised mitochondria can also contribute to cellular death pathways. During infection, host-derived stresses, including plant phytoalexins and oxidative bursts, can damage fungal mitochondria, causing membrane dysfunction and potentially cell death [29]. Hemibiotrophic pathogens, such as *M. oryzae*, generally require host cells to remain alive during the biotrophic phase and therefore deploy effectors that modulate host defense and programmed cell death [30,31]. Necrotrophs such as *B. cinerea*, in contrast, promote host cell death through mechanisms that include toxin production and NADPH oxidase-derived ROS, while also requiring mechanisms that protect fungal cells from the resulting oxidative and metabolic stresses. In *B. cinerea*, genetic enhancement of anti-apoptotic capacity suppressed host-triggered PCD and increased virulence, but compromising this capacity reduced pathogenicity [32,33].

Fungi employ flexible regulatory networks to control cell-death pathways, involving apoptosis-inducing factors, inhibitors of apoptosis proteins, and metacaspases [29,34]. Through these interconnected mechanisms, mitochondria function not only as bioenergetic and biosynthetic hubs but also as important regulators of cellular stress and fate during infection [7]. As illustrated in Figure 1, fungal pathogenicity depends on maintaining a delicate balance between mitochondrial functions that support growth and pathogenicity and those that trigger oxidative damage and programmed cell death. Therefore, mitochondria’s ability to maintain functional metabolism while limiting damage is a fundamental requirement for fungal adaptation to the hostile host environment and provides important biological context for exploring mitochondrial quality control mechanisms.

**Figure 1.**
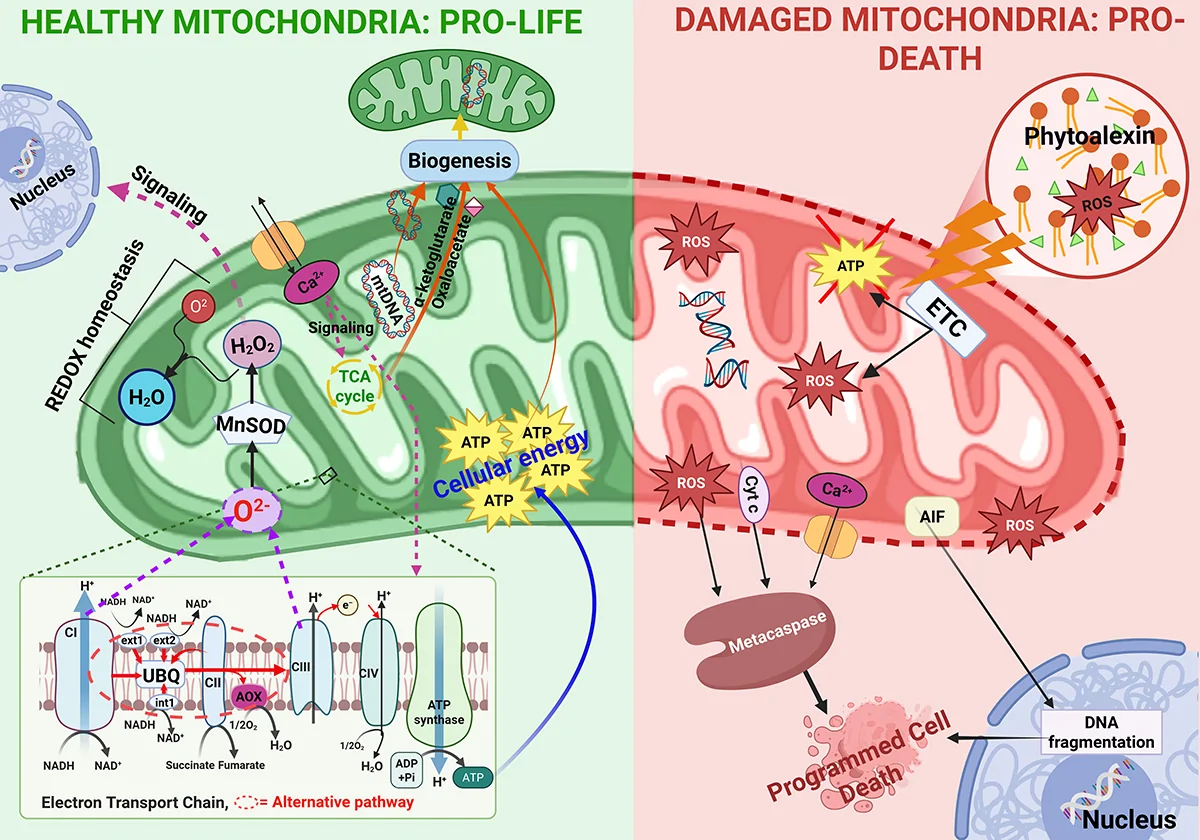
Mitochondria as cellular hubs of life and death. Beyond their canonical and pivotal role in energy production, mitochondria also integrate stress signals and regulate metabolism and homeostasis. They serve as both sources and targets of ROS, and ROS targeting can damage their integrity and activate mitochondrial-dependent programmed cell death pathways. ATP: adenosine triphosphate, ROS: reactive oxygen species, etc: electron transport chain, mtDNA: mitochondrial DNA, TCA: tricarboxylic acid, AOX: alternative oxidase, AIF: apoptosis-inducing factor, MnSOD: manganese superoxide dismutase. Created in BioRender. TOUKAP, H. (2026) https://biorender.com/c79abde.

#### Mechanisms of MQC in phytopathogenic fungi

To adapt to stress and protect their essential yet vulnerable mitochondria, phytopathogenic fungi possess an integrated mitochondrial quality control system with interconnected mechanisms that operate across different levels of mitochondrial damage and dysfunction, from protein-level repair and organelle remodeling to selective removal of damaged mitochondria. These mechanisms can be broadly grouped into mitochondrial dynamics, biogenesis, proteostasis, and mitophagy, which collectively contribute to mitochondrial homeostasis and support infection (Figure 2).

**Figure 2.**
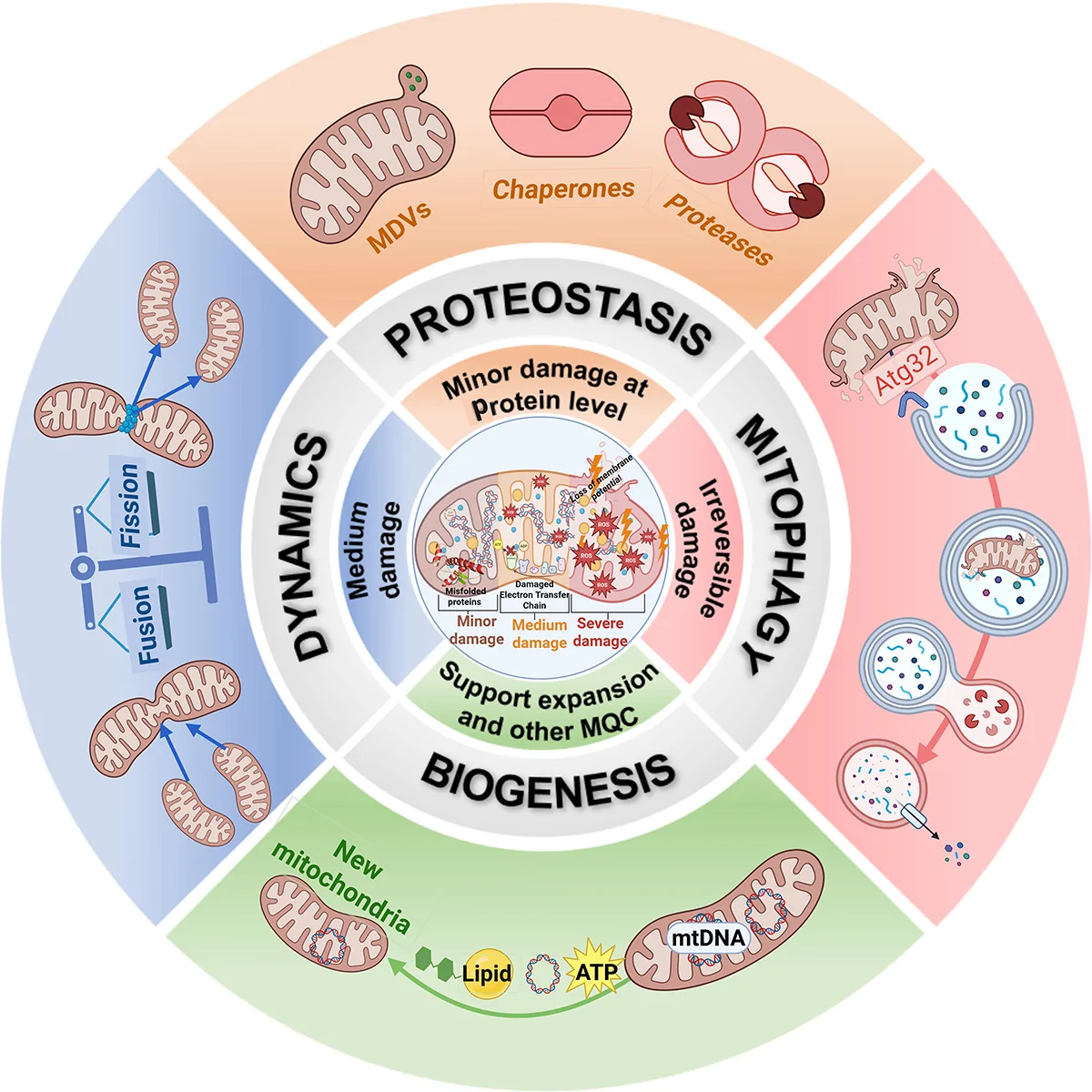
The mitochondrial quality control (MQC) network in phytopathogenic fungi. MQC comprises four interconnected layers that preserve mitochondrial integrity and functional capacity. (1) Proteostasis maintains mitochondrial protein quality through chaperones, proteases, protein-import surveillance, and mitochondrial-derived vesicles. (2) Mitophagy selectively removes dysfunctional mitochondria. (3) Biogenesis replenishes mitochondrial components and supports the expansion and renewal of the mitochondrial network. (4) Dynamics continuously remodels the mitochondrial network through fission and fusion, enabling redistribution of organelles and segregation of damaged components. Created in BioRender. TOUKAP, H. (2026) https://biorender.com/poq9apx.

### Mitochondrial dynamics: the continuous remodeling of the network

Mitochondrial dynamics refer to the regulated, continuous remodeling of the organellar network through the opposing yet complementary processes of fission and fusion [16]. Fission divides a single mitochondrion into separate units, which is essential for creating new organelles, distributing them throughout the cell, and, more importantly, isolating damaged segments for repair or disposal. In yeast, four main proteins are involved in the fission machinery: the outer mitochondrial membrane protein Fis1 and the cytosolic proteins Dnm1, Mdv1, and Caf4 [35]. Among pathogenic fungi, cytosolic Dnm1 is relatively conserved and drives mitochondrial fission in *M. oryzae*, in complex with the adaptor MoMdv1 and the membrane anchor MoFis1. In *M. oryzae*, rapid mitochondrial proliferation is required to fuel appressorial turgor generation; therefore, deleting MoDnm1 significantly reduced pathogenicity [13,36]. In *Fusarium graminearum*, the fission protein FgDnm1 influenced mitochondrial morphology and toxin production during the necrotrophic phase, and its deletion attenuated virulence [37]. This suggests that while fission machinery is relatively conserved, its contribution to pathogenicity depends on the pathogen’s lifestyle.

Fusion merges two mitochondria and mixes their contents, including proteins, lipids, and mtDNA, which dilutes local damage and supports mitochondrial function. The fusion machinery is relatively conserved across the animal and fungal kingdoms, involving mitofusins such as Fzo1 for outer-membrane fusion and a dynamin-related GTPase like Mgm1 (the homolog of mammalian OPA1) for inner-membrane fusion [38]. In pathogenic fungi such as *Aspergillus fumigatus*, *Cryptococcus neoformans* and *M. oryzae*, the deletion of Fzo1 or Mgm1 resulted in fragmented networks and significantly reduced virulence, supporting an important role for mitochondrial fusion in pathogenicity [13,39,40].

Moreover, some pathogen-specific regulators control the fission-fusion balance to support organellar functions. In *M. oryzae*, the deubiquitinating enzymes encoding genes MoUbp12 and MoUbp14, modulated by the transcription factor MoMsn2, are critical for maintaining this balance; their deletion resulted in excessive and dysregulated fission and fusion, leading to reduced infectious growth [41]. This specialized regulation ensures that the mitochondrial network can meet the unique demands of plant infection, including polarized growth and stress adaptation during host-cell penetration.

### Mitochondrial biogenesis, import, and layered turnover

Mitochondrial biogenesis is a coordinated process involving the expression, targeting, import, and assembly of proteins encoded by both the nuclear and mitochondrial genomes [42]. Most nuclear-encoded proteins are synthesized in the cytosol and imported through specialized pathways, with TOM serving as the main entry gateway; subsequent sorting involves SAM/TOB, MIM, TIM23, TIM22, MIA, and Oxa1, depending on protein type (β-barrel proteins, α-helical proteins) and destination. Together, these pathways ensure accurate mitochondrial protein targeting and assembly, thereby supporting the maintenance of functional mitochondria [43–45]. To manage molecular damage, mitochondria use a layered defense system: the first line is intra-organellar proteostasis, involving resident chaperones such as Hsp60 and ATP-dependent proteases that refold or degrade misfolded proteins [46]. In *B. cinerea*, the mitochondrial Lon1 protease, an ATP-dependent protease, degrades misfolded proteins in the matrix and is essential for maintaining mitochondrial homeostasis under heat stress. Deletion of Bclon1 induced mitochondrial dysfunction and accelerated cell death, and those phenotypes were exacerbated when autophagy was simultaneously inhibited, indicating functional coordination between proteostasis and autophagic pathways [47]. Another selective process involves mitochondrial-derived vesicles, which bud off to deliver damaged cargo directly to vacuoles for degradation, enabling piecemeal repair without compromising the entire organelle [48]. When constitutive repair is overwhelmed, fungi activate nucleus-mediated stress responses, including the fungal unfolded protein response and the mitochondrial compromised protein import response, to detect specific failures, such as import defects, and initiate transcriptional programs to restore mitochondrial homeostasis [46].

### Mitophagy: the clearance mechanism

Mitophagy is the selective autophagic removal of damaged or dysfunctional mitochondria and represents an important mitochondrial quality control mechanism for limiting the accumulation of defective organelles and associated oxidative stress [12]. In the canonical yeast mitophagy pathway, outer membrane receptors such as Atg32 recognize and recruit autophagic machinery to depolarized or damaged mitochondria, thereby promoting their sequestration and delivery to the vacuole for degradation. In *M. oryzae*, deletion of MoAtg24 disrupted mitophagy in foot cells and significantly reduced conidiation [49].

Moreover, mitophagy is functionally integrated with other MQC layers; damaged mitochondria may be repaired through proteostasis, redistributed through fusion, or selectively eliminated through mitophagy, depending on the nature and extent of mitochondrial damage. Mitochondrial fission can facilitate mitophagy by segregating damaged regions and generating smaller mitochondrial units that can be selectively targeted for autophagic removal [50]. Together, these interconnected processes link mitochondrial repair, remodeling, and turnover, which contribute to mitochondrial homeostasis and, in documented phytopathogenic fungi, support infection-related development and pathogenicity [14,42]. Therefore, understanding how these coordinated MQC mechanisms are deployed at specific stages of the infection cycle may reveal vulnerabilities that could be leveraged for disease management.

#### Vulnerability of MQC and targeting opportunities

Fungal infection is a dynamic process involving successive developmental, metabolic, and physiological transitions. Each transition imposes distinct demands on mitochondrial function, requiring the pathogen to continuously remodel, repair, replenish, and eliminate mitochondrial components while maintaining sufficient bioenergetic and redox capacity [5,13]. The resulting dependence places mitochondrial quality control (MQC) at the intersection of mitochondrial homeostasis and pathogenicity. Importantly, the contribution of MQC is not uniform across the infection cycle or among fungal lifestyles; most of the mechanistic evidence linking MQC to infection stage transitions comes from the rice blast fungus *M. oryzae*, as it is the most characterized phytopathogenic fungus to date, and emerging evidence from other fungi reveals both conserved and lifestyle-specific requirements. The evidence is organized into two complementary levels: direct MQC evidence, including experimentally demonstrated roles for mitochondrial dynamics, biogenesis, proteostasis, or selective turnover in fungal infection, and complementary mitochondrial competence evidence that is not a MQC mechanism itself but instead outlines the mitochondrial functions that MQC must preserve for successful infection. This distinction allows evaluation of the pathogenic role of MQC without equating every mitochondrial phenotype with a direct MQC function.

To provide an integrated view of the available evidence, Table 1 summarizes experimentally characterized mitochondrial quality-control (MQC) mechanisms and complementary mitochondrial functions associated with fungal development and pathogenicity across representative phytopathogenic fungi. Because the strength and type of evidence vary substantially among studies, the table distinguishes direct MQC evidence from MQC-associated processes and complementary mitochondrial-function evidence. This distinction matters because defects in mitochondrial respiration, metabolism, transport, or redox homeostasis show loss of mitochondrial functional competence but do not, by themselves, establish a direct role for an MQC pathway.

**Table 1.** Evidence linking mitochondrial quality control to pathogenicity in phytopathogenic fungi.

Fungal species	MQC component	MQC layer	Infection stage	Phenotypic consequence	Evidence category	Evidence basis	Reference
*M. oryzae*	MoDnm1/MoFis1/MoMdv1	Dynamics	Appressorium, penetration	Impaired appressorium function and penetration	Direct MQC	Genetic/mechanistic	[13,36]
*M. oryzae*	MoFzo1	Dynamics	Colonization	Altered mitochondrial morphology and impaired invasive growth	Direct MQC	Genetic	[13]
*M. oryzae*	MoAtg24	Mitophagy	Conidiation, Colonization	Defective mitochondrial clearance and reduced infection	Direct MQC	Genetic/mechanistic	[13]
*M. oryzae*	MoAti1	Mitophagy	Colonization	Reduced mitophagy and invasive growth	Direct MQC	Genetic/mechanistic	[51]
*C. camelliae*	CaSun1	Mitophagy	Colonization	Reduced mitophagy and pathogenicity	Direct MQC	Genetic/mechanistic	[52]
*V. dahliae*	VdATG24	Mitophagy	Colonization	Reduced growth, microsclerotia and virulence	Direct MQC	Genetic/mechanistic	[53]
*T. roseum*	TrAtg8-mediated mitophagy	Mitophagy	Early infection	Mitochondrial dysfunction, reduced energy charge, alkalinization and pathogenicity	Direct/MQC-associated	Pharmacological/mechanistic	[54]
*F. graminearum*	FgDnm1	Dynamics	Invasive growth, toxin production	Altered mitochondrial dynamics and DON production	Direct MQC	Genetic	[37]
*B. cinerea*	BcMtp1	Mitochondrial competence	Colonization	Disrupted mitochondrial homeostasis and virulence	MQC-associated	Genetic	[55]
*B. cinerea*	BcAOX	Respiration	Colonization	Reduced sporulation/virulence and altered oxidative stress	Complementary mitochondrial function	Genetic	[21]
*B. cinerea*	Bccox15	Respiration/mitochondrial competence	Sporulation, Colonization	Altered respiration, oxidative homeostasis, spore longevity and virulence	Complementary mitochondrial function	Genetic	[56]
*C. camelliae*	CaEch1	β-oxidation/mitophagy-associated phenotype	Germination, appressorium	Reduced development and pathogenicity	MQC-associated/complementary	Genetic	[57]
*V. dahliae*	VdNuo1	Respiration	Colonization	Impaired mitochondrial function and virulence	Complementary mitochondrial function	Genetic	[58]
*V. dahliae*	VdAtuA3	Mitochondrial regulatory/homeostatic function	Colonization	Perturbed mitochondrial homeostasis and reduced pathogenicity	Complementary mitochondrial function	Genetic, pharmacological	[59]
*T. roseum*	TrSnt2	Mitophagy-associated regulation	Colonization	Altered mitophagy, ROS accumulation, ergosterol synthesis, and pathogenicity	MQC-associated	Genetic, mechanistic	[60]
*M. oryzae*	MoCox6	Mitophagy	Colonization	Altered mitophagy and reduced pathogenicity; pharmacological targeting reported	Direct MQC	Genetic, pharmacological	[61,62]
*M. oryzae*	MoTom20	Mitochondrial organization/dynamics	Appressorium, Colonization	Perturbed mitochondrial organization and infection-related development	MQC-associated	genetic	[63]

### Pre-penetration: spore quality and germination

The transition from a dormant spore to a metabolically active germling represents one of the earliest mitochondrial challenges in the fungal infection cycle. Before external nutrients become available, germinating spores rely heavily on endogenous reserves and on the functional mitochondrial population inherited during sporulation [64]. Consequently, mitochondrial quality established during spore formation can influence the next generation’s capacity to germinate and initiate infection. In *M. oryzae*, live-cell imaging has shown that tubular mitochondria are actively transported into developing conidia and undergo fission during septum formation, generating the discrete mitochondrial population inherited by the mature spore [64]. Disruption of the class II myosin Momyo2 interfered with conidiation and produced spores with germination defects, illustrating the importance of coordinated organelle inheritance during spore development [65]. Furthermore, mitophagy contributed to the quality of this inherited mitochondrial population; in conidiophore foot cells, deletion of the mitophagy regulator MoAtg24 disrupted mitochondrial clearance, reduced conidial viability, and increased sensitivity to oxidative stress [49]. Likewise, the stress-response protein MoWhi2 was found to be specifically required for mitophagy in *M. oryzae* foot cells, and its deletion disrupted mitophagy and reduced conidiation [66]. Together, these observations establish a mechanistic connection between mitochondrial turnover during sporulation and the quality of the mitochondrial pool available for subsequent germination.

The requirement for mitochondrial competence continues after spore maturation; germination requires rapid mobilization of endogenous carbon reserves and activation of mitochondrial metabolism. In *M. oryzae*, deletion of the mitochondrial β-oxidation component Ech severely reduced germination [67]. β-oxidation itself is not an MQC mechanism; rather, this study demonstrates the functional consequence of failing to maintain functional mitochondria capable of supporting the metabolic transition from dormancy to active growth. Thus, the pre-penetration vulnerability involves two connected processes: MQC maintains an appropriate mitochondrial population during sporulation, while mitochondrial metabolic competence enables that population to germinate. This dependence is also evident outside *M. oryzae*; in the necrotroph *B. cinerea*, the mitochondrial heme-A synthase Bccox15 contributes to respiratory and oxidative homeostasis, and its deletion compromises conidial formation, viability over time, germination, and pathogenicity. The mutant exhibits altered mitochondrial morphology, reduced membrane potential, and increased oxidative stress [56]. Although not a MQC component, Bccox15 provides complementary evidence that mitochondrial functional integrity is important for maintaining the developmental and pathogenic capacity of spores.

Mitochondrial dysfunction can also be exploited chemically during this stage; plant-derived compounds, including perillaldehyde, 2-phenylethanol, and cuminaldehyde, inhibit germination in *B. cinerea* and *Penicillium digitatum* through mitochondrial membrane depolarization and oxidative stress [68–70]. While surfactin induced mitochondrial ROS and cell-death responses in *F. graminearum* germlings [71]. More recently, cuminal-triggered mitochondrial dysfunction in *Tricothecium roseum* was shown to excessively induce TrAtg8-mediated mitophagy, leading to reduced energy charge in spores [54]. These studies demonstrate that disturbing mitochondrial homeostasis during early development can compromise fungal fitness across distinct pathogenic lifestyles.

Thus, the pre-penetration stage represents a vulnerability created by spores’ dependence on a carefully maintained mitochondrial population. Direct MQC studies highlight the importance of mitochondrial inheritance and selective turnover, while complementary mitochondrial-function studies demonstrate the consequences of losing respiratory, energetic, or redox competence. Together, they establish mitochondrial quality as an important determinant of the transition from spore survival to active infection.

### Penetration: the breach of Host defenses

Successful penetration requires fungi to overcome the physical and chemical barriers of the plant surface. The strategy used differs substantially among pathogenic lifestyles, ranging from mechanical penetration through specialized infection structures to enzymatic degradation of host tissues and exploitation of natural openings [72]. These differences impose distinct demands on cellular bioenergetics and, consequently, on MQC.

#### The appressorium: a paradigm of force and MQC coordination

The appressorium provides the most extensively characterized example of mitochondrial adaptation during fungal penetration. In *M. oryzae* and other appressorium-forming pathogens such as *Colletotrichum higginsianum*, the infection structure undergoes a major metabolic and morphological transition before generating the high turgor pressure (≥8 MPa) required to breach the host cuticle [73,74]. In *M. oryzae*, MoDnm1, MoFis1, and MoMdv1 are important for mitochondrial fission and mitophagy and are required for appressorium development. Loss of MoDnm1 produces abnormal mitochondrial morphology and impairs functional appressorium formation and host penetration. Treatment with the mitochondrial fission inhibitor Mdivi-1 resulted in similar mitochondrial morphology defects; specifically, fewer mitochondrial punctate patterns were observed, with reduced pathogenicity on barley leaves [36]. Additionally, another study showed that Mrm1, a putative rRNA methyltransferase, contributes to the balance of mitochondrial fission and fusion and helps generate turgor pressure. Deletion of Mrm1 in *M. oryzae* delayed appressorium formation, reduced turgor pressure for host penetration, and infection hyphae expansion [75]. Furthermore, mitochondrial biogenesis and protein import also contribute to this phase. In *M. oryzae*, mutants lacking the outer membrane translocase MoTom20 display punctate mitochondria, loss of membrane potential, and a critical turgor defect, causing appressoria to initiate but fail to complete penetration, directly linking mitochondrial biogenesis to the mechanical power output of the infection cell [63].

Beyond rice blast, studies in *Colletotrichum camelliae* showed that the mitochondria-localized enoyl-CoA hydratase CaEch1 is required for mitophagy, and CaECH1 deletion impairs conidial germination and appressorium development, linking mitochondrial quality control to infection structure formation [57]. A complementary example is *Verticillium dahliae*, where VdATG24 functions as a mitophagy receptor and contributes to fungal growth and virulence. Mechanistic analyses further associate VdATG24 with melanin biosynthesis and protein secretion during infection, providing a potential bridge between selective mitochondrial turnover and the secretory demands of host colonization [53]. The appressorium therefore represents a clear example of how MQC supports an infection-associated metabolic transition; mitochondrial fission and organelle remodeling must accommodate a sudden increase in metabolic demand. The resulting dependence creates an infection-stage vulnerability relatively well documented in *M. oryzae* but may extend to other appressorium-forming pathogens.

#### Alternative penetration strategies

Appressorium-mediated penetration is not universal; necrotrophic fungi such as *B. cinerea* and *T. roseum* rely heavily on extracellular enzymes, toxins, organic acids or bases, and oxidative mechanisms to damage host tissues, whereas *Sclerotinia sclerotiorum* combines mechanical force with concentrated secretion through infection cushions [76–79]. Rust fungi such as *Puccinia striiformis* can exploit natural openings and therefore do not require the same high-turgor penetration mechanism [80]. These differences provide an important comparative perspective: the mitochondrial requirement is conserved, but the infection process that generates the requirement is lifestyle-specific.

For necrotrophs, tissue invasion depends on sustained production, folding, trafficking, and secretion of large quantities of extracellular virulence factors. This imposes demands on ATP production, biosynthetic precursor supply, redox balance, and intracellular protein homeostasis. At the same time, necrotrophs frequently generate ROS as part of their attack strategy and are exposed to ROS produced by the host, producing a ROS-rich environment at the infection interface that threatens the pathogen’s own mitochondria [78,81,82]. These organelles are a primary site of antioxidant production, housing enzymes such as mitochondrial superoxide dismutase, which are essential for detoxifying ROS and maintaining virulence [58]. Virulence, therefore, depends not only on the capacity to generate this oxidative weapon but also on maintaining a healthy mitochondrial population capable of producing sufficient antioxidants to neutralize ROS from both sources and prevent self-inflicted oxidative damage.

MQC can therefore support enzymatic invasion indirectly by maintaining the mitochondrial metabolic and redox capacity required for sustained production of virulence-associated factors under oxidative stress. In *T. roseum*, the phospholipase D TrPLD3 provides complementary evidence that infection is accompanied by substantial oxidative and membrane-associated demands. TrPLD3 participates in phosphatidic acid metabolism and promotes NADPH oxidase-dependent ROS production, thereby contributing to host tissue damage and pathogenicity [83]. Although TrPLD3 is not a mitochondrial MQC component, its role in regulating ROS and membrane lipid signaling highlights the oxidative environment in which mitochondrial homeostasis must be maintained during necrotrophic invasion, justifying the requirement for mitochondrial quality control. More direct evidence comes from recent work on *T. roseum*. Cuminal treatment induced oxidative stress, mitochondrial depolarization, altered mitochondrial dynamics, and TrAtg8-mediated mitophagy. The resulting excessive mitophagy decreased energy charge by approximately 44%, suppressed expression of cell-wall-degrading enzymes, impaired environmental alkalinization, and reduced pathogenicity [54]. These findings support a link between mitochondrial quality-control imbalance, energetic capacity, virulence-factor expression, and host colonization. Moreover, the study also demonstrates that excessive mitophagy can be detrimental to cellular growth, indicating that pathogenicity depends on appropriately calibrated mitochondrial turnover. These findings do not establish that mitophagy directly controls cell-wall-degrading enzyme secretion, but they strengthen the emerging connection between MQC, mitochondrial fitness, secretion, and pathogenic development in non-appressorium-forming pathogens.

### Post-penetration: initial establishment and colonization

Following penetration, pathogens must establish a stable foothold within the host’s living or dying tissue while adapting to nutrient limitation, host-derived oxidative stress, and changing metabolic conditions [33]. This stage provides strong evidence that MQC is not simply required for fungal survival but is actively coordinated with infection development. For biotrophic and hemibiotrophic pathogens, this involves establishing sustained intracellular growth while maintaining compatibility with living host cells [30]. For necrotrophs, by contrast, successful establishment requires rapid host killing, detoxification, and continued metabolic adaptation during tissue colonization [33].

*M. oryzae* illustrates the importance of mitochondrial remodeling during invasive growth; following mechanical penetration, the penetration peg expands into primary invasive hyphae within the first living rice cell [31]. During this transition, mitochondrial morphology undergoes a characteristic filamentous-to-punctate-to-filamentous cycle. Disrupting either mitochondrial fusion or fission by deleting MoFzo1 or MoDnm1 reduced pathogenicity, while pharmacological inhibition of mitochondrial fission with Mdivi-1 produced similar defects [13]. Although Mdivi-1 is not a definitive Dnm1-specific inhibitor, these genetic and pharmacological observations together indicate that dynamic mitochondrial remodeling supports invasive growth.

Mitophagy also contributes to mitochondrial homeostasis during the invasive phase of *M. oryzae* infection. MoAtg24-mediated mitophagy is required for proper biotrophic development, while MoAti1 contributes to mitochondrial clearance in invasive hyphae during pathogenesis. Deletion of either gene reduces pathogenicity *in planta* [13,51]. Together, these findings provide a mechanistic model in which mitochondrial dynamics and selective mitochondrial turnover are coordinated to maintain mitochondrial competence during host colonization. In addition, the transcription factor MoMsn2 regulates mitochondrial fusion and fission through its target MoAuh1; both ΔMomsn2 and ΔMoauH1 mutants exhibit reduced pathogenicity and defective infectious hyphal growth [84]. Thus, mitochondrial remodeling during invasive growth is controlled at both the organelle and regulatory levels.

This MQC–pathogenicity relationship extends beyond *M. oryzae*; in *Colletotrichum camelliae*, the SUN-family protein CaSun1 localizes to mitochondria, recruits CaAtg8, and promotes mitophagy during nitrogen starvation. Loss of CaSun1 compromises invasive hyphal growth and pathogenicity [52]. In *Colletotrichum higginsianum*, the prohibitin complex ChPhb1/ChPhb2 regulates mitochondrial integrity and interacts with the mitophagy receptor ChAtg24. Disruption of the prohibitin complex or ChAtg24 blocks mitophagy and impairs the transition to necrotrophic growth and the formation of secondary hyphae [85]. Further evidence comes from the vascular pathogen *V. dahliae*. VdATG24 contains characteristic domains associated with membrane recognition and Atg8 interaction and functions as a component of prohibitin-mediated mitophagy. Its deletion affected fungal growth, conidiation, microsclerotia formation, and host colonization [53]. Direct evidence linking mitophagy and pathogenicity has been established in pathogens occupying different ecological niches, strengthening the conclusion that MQC–pathogenicity coupling is not a peculiarity of the rice blast fungus.

For necrotrophic pathogens, establishment and colonization impose different mitochondrial demands because successful infection depends on rapid host killing, detoxification, and sustained tissue degradation. In *Botrytis cinerea*, the mitochondrial transporter BcMtp1 is required for metabolic homeostasis; its deletion causes defects in growth and stress tolerance and abolishes lesion formation [55]. Although BcMtp1 is not an MQC component, this phenotype provides complementary evidence that mitochondrial transport and functional competence are required to sustain the metabolic, stress-response, and invasive processes underlying necrotrophic expansion.

In *Trichothecium roseum*, the epigenetic reader TrSnt2 provides additional evidence of a link between transcriptional regulation and mitochondrial quality control during pathogenesis. Deletion of Trsnt2 increased H3 acetylation, upregulated multiple autophagy-related genes, and promoted mitophagy. However, this enhanced mitophagy was accompanied by disrupted ROS homeostasis, including altered expression of ROS-producing and ROS-scavenging genes, impaired ergosterol biosynthesis, and membrane damage. These combined defects markedly reduced pathogenicity on muskmelon and tomato fruits [60]. These findings suggest that mitochondrial turnover must remain coordinated with redox and membrane homeostasis to preserve cellular integrity during necrotrophic infection. Overall, the post-penetration phase provides strong evidence for the functional coupling between mitochondrial quality control and pathogenicity across various pathosystems.

### Sustained colonization and secondary spread

Once established within the host tissue, the pathogen enters a prolonged phase of sustained colonization, facing ongoing challenges such as nutrient acquisition, competition, evasion of host defenses, and ultimately, reproduction for secondary dispersal [86]. MQC becomes particularly important during this prolonged phase because mitochondrial damage accumulates, but the pathogen must preserve sufficient organelle function for continued growth and reproduction.

During sustained colonization, pathogens must maintain metabolic output under prolonged stress. Here, selective mitophagy serves as a crucial MQC mechanism that supports long-term virulence. In *F. graminearum*, inhibiting mitophagy significantly reduces mycelial biomass, conidiation, and deoxynivalenol toxin synthesis [87]. These phenotypes show that selective mitochondrial turnover supports sustained fungal development and secondary spread. In *C. higginsianum*, disruption of the prohibitin–ChAtg24 mitophagy system prevents the transition to necrotrophic growth and blocks secondary hyphae formation, directly linking mitochondrial quality control to the developmental program responsible for tissue maceration and continued colonization [85].

Mitochondrial dynamics also intersect with specialized metabolism and fungicide response. In *F. graminearum*, FgDnm1 regulates mitochondrial morphology and contributes to toxisome formation and deoxynivalenol production; mitochondrial fragmentation induced by QoI and SDHI fungicides is altered in the ΔFgDnm1 mutant, showing that mitochondrial remodeling influences the fungal response to respiratory inhibitors [37]. This observation provides a mechanistic bridge between MQC status and chemical sensitivity.

Mitochondrial competence provides complementary evidence: in *B. cinerea*, BcMtp1 is required for metabolic homeostasis, stress tolerance, conidiation, and survival structure formation [55]. In *V. dahliae*, VdNuo1 supports mitochondrial integrity, stress adaptation, and microsclerotia development [88]. These examples are not MQC mechanisms, but they show that long-term pathogenicity requires a mitochondrial network that can sustain both metabolism and developmental transitions, which MQC is meant to preserve.

Reproductive development provides a final MQC-dependent transition; in *M. oryzae*, MoAtg24-mediated mitophagy and MoWhi2-dependent mitochondrial quality control contribute to conidiation [13,66]. In *B. cinerea*, Bccox15 provides complementary evidence that maintaining mitochondrial respiratory and oxidative homeostasis is necessary for spore longevity, germination, and virulence [56]. Together, these findings indicate that mitochondrial quality influences not only immediate infection success but also the production and fitness of secondary inoculum.

Taken together, the evidence indicates that mitochondrial and MQC requirements are not uniform throughout the fungal infection cycle. Instead, different mitochondrial processes become particularly important during germination, host penetration, invasive growth, and reproduction, although evidence strength varies among pathogens and processes. This temporal organization is best resolved in appressorium-forming hemibiotrophic fungi, particularly *M. oryzae*, and is summarized in Figure 3.

**Figure 3.**
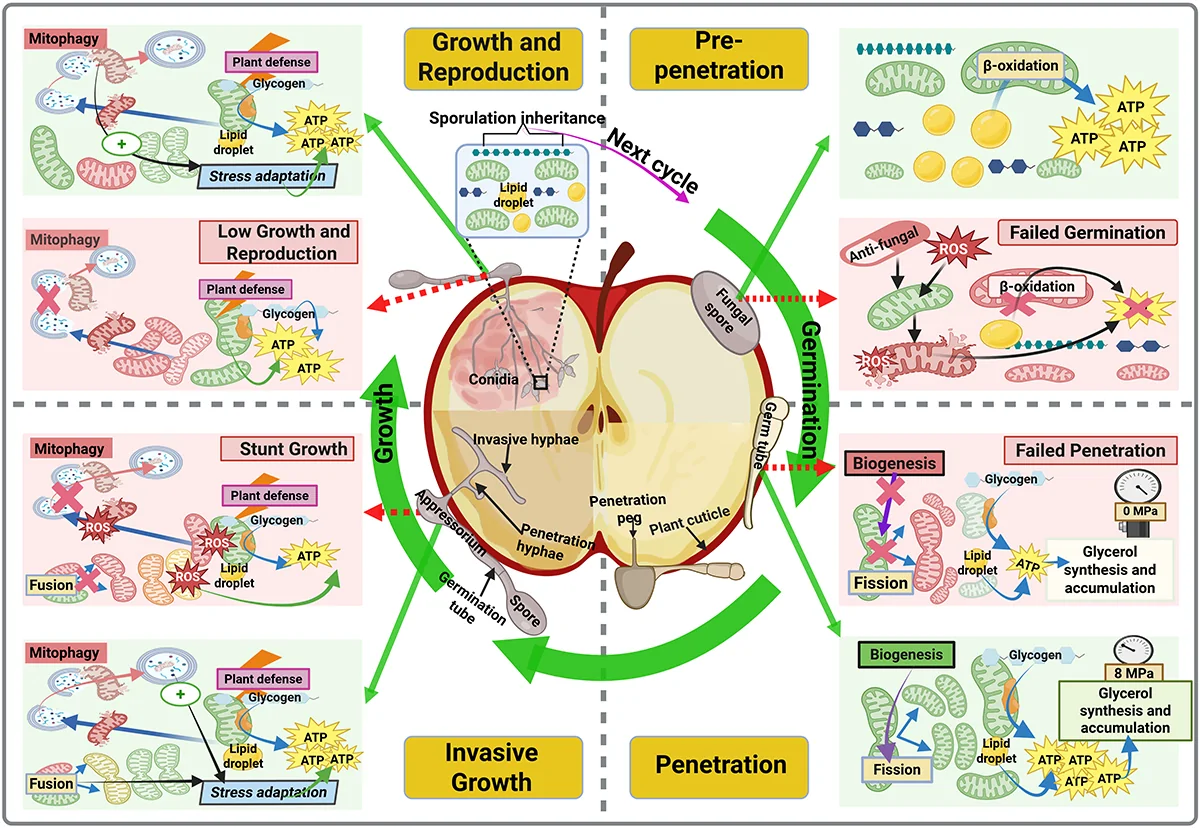
Stage-specific mitochondrial and mitochondrial quality-control (MQC) dependencies during infection in appressorium-forming fungi. This figure illustrates how mitochondrial functional requirements and MQC mechanisms contribute to successive stages of infection, using *Magnaporthe oryzae* and related appressorium-forming pathogens as the principal experimental framework. (1) Pre-penetration: mitochondrial metabolic competence, including β-oxidation, supports conidial germination and developmental transitions; β-oxidation represents mitochondrial physiology rather than a core MQC mechanism. (2) Penetration: mitochondrial biogenesis and fission support the high bioenergetic demand associated with appressorium maturation and host penetration. (3) Invasive growth: mitochondrial dynamics and selective mitophagy contribute to maintenance of mitochondrial fitness during sustained host-associated stress in selected pathogens. (4) Reproduction: mitophagy-associated mechanisms and mitochondrial homeostasis contribute to conidiation and spores’ fitness. Green indicates experimentally supported functional requirements; red indicates developmental or infection defects following genetic or pharmacological perturbation. The figure illustrates stage-specific biological dependencies and potential intervention points. Created in BioRender. TOUKAP, H. (2026) https://biorender.com/5q2p0r5.

#### Exploiting MQC vulnerabilities for disease control

MQC operates as an interconnected maintenance network whose requirements vary with fungal development, environmental stress, and infection stage. This stage dependence does not establish a therapeutic window on its own, but it identifies biological contexts in which perturbing mitochondrial quality or competence may disproportionately affect fungal performance. Translating these dependencies into disease-control strategies requires two complementary considerations: achieving selectivity against the pathogen despite relative conservation of core MQC machinery, and overcoming the robustness and interdependence of mitochondrial quality-control networks [5,15]. The available evidence supports three complementary intervention dimensions (Figure 4). First, pathogen-specific regulators and regulatory interfaces may provide selective targets within conserved mitochondrial pathways. Second, interdependence among MQC processes and mitochondrial stress responses may create combinatorial vulnerabilities that rational combinations can exploit. Third, stage-specific mitochondrial requirements may guide intervention timing when experimental data show differential sensitivity. Together, these dimensions connect mechanistic MQC biology with target validation and the practical requirements of antifungal development.

**Figure 4.**
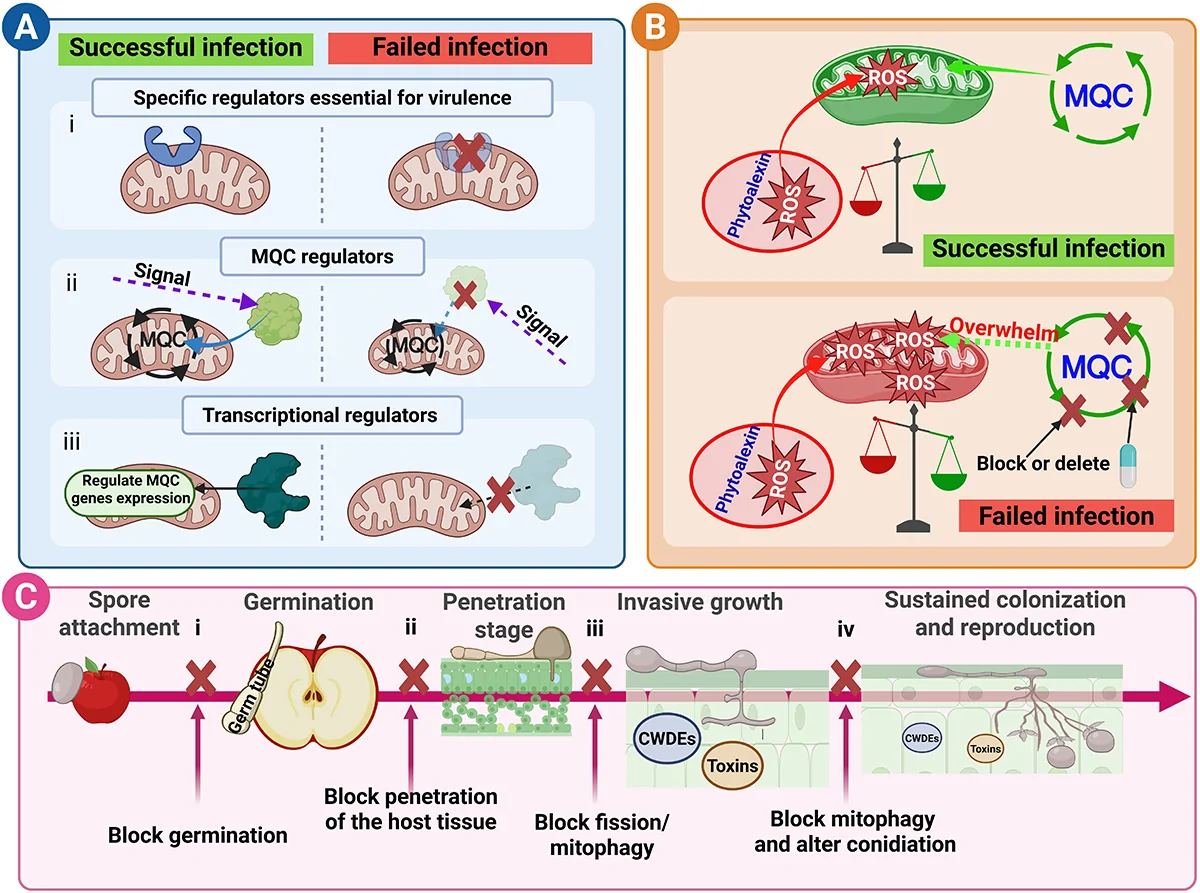
Antifungal paradigm targeting vulnerabilities in mitochondrial quality control (MQC) in phytopathogenic fungi. (A) Targeting selective vulnerabilities within the fungal mitochondrial quality control signaling hierarchy. (i) Context-specific regulatory proteins essential for MQC during specific infection stages. (ii) Signaling hub nodes integrate upstream inputs to fine-tune MQC activity; their disruption collapses stress responses. (iii) Master transcriptional regulators controlling MQC-related genes during infection. (B) Exploiting MQC network interconnections. Intimate connections between MQC processes create exploitable vulnerabilities. Simultaneous disruption of interconnected pathways may overwhelm homeostatic capacity and shift the balance toward oxidative stress. (C) Phase-tailored interventions counteracting the pivotal roles of MQC during the fungal infection cycle. (i) Blocking mitochondrial metabolism during germination halts pre-penetration development. (ii) Suppressing biogenesis/fission during the penetration stage. (iii) Disrupting fission/fusion or mitophagy during invasive growth affects colonization. (iv) Blocking mitophagy during reproduction impairs conidiation and spore quality, breaking the disease cycle. Created in BioRender. TOUKAP, H. (2026) https://biorender.com/y0u5bm6.

### Selective vulnerabilities within MQC regulatory hierarchies

A key consideration when evaluating MQC components as antifungal targets is the distinction between housekeeping-essential functions and infection-preferential functions. Disruption of a core mitochondrial process may reduce pathogenicity simply because it severely compromises general fungal growth or viability, whereas a regulator that has a disproportionate effect during host infection may offer a more attractive anti-virulence target [89]. Accordingly, MQC-related factors can be viewed along a functional continuum comprising housekeeping-essential components, general fitness determinants, infection-preferential regulators, and more specialized virulence-associated factors. The therapeutic value of a candidate target therefore depends not only on whether its disruption reduces virulence, but also on the magnitude and context of the infection defect relative to its effect on basal fungal fitness. This distinction matters for the proposed “disarm” strategy: targets that impair infection disproportionately while preserving limited vegetative fitness may offer greater scope for selective disease suppression than targets whose inhibition simply causes broad fungal toxicity.

Context-specific regulators represent an important example; in *M. oryzae*, MoAti1 promotes recruitment of MoAtg8 to mitochondria and is required for mitochondrial turnover during invasive growth. Disrupting MoAti1 reduces pathogenicity, while host-induced gene silencing of MoATI1 enhances resistance to rice blast [51,52]. The absence of a clear MoAti1 counterpart in rice may support pathogen selectivity; however, genetic and host-induced silencing alone cannot establish it as a chemical target. Similarly, CaSun1 promotes mitophagy in *C. camelliae* by recruiting CaAtg8, and its disruption compromises invasive growth and pathogenicity [52]. Because SUN-family proteins are conserved, the CaSun1 example also illustrates why fungal-versus-host selectivity cannot be inferred simply from a protein’s contribution to virulence. Instead, more research is needed to experimentally identify pathogen-specific structural features, regulatory interactions, or binding interfaces. A recent study of *Verticillium dahliae* provides a particularly strong example of exploiting a pathogen-specific mitochondrial regulatory interface. VdAtuA3, a mitochondrial regulator, interacts with the highly conserved respiratory-chain component VdNuo1. VdAtuA3 shows low sequence similarity to corresponding proteins from plants and animals, and yeast two-hybrid assays detected no interaction between Nuo1 and AtuA homologs from *Gossypium hirsutum*, *Nicotiana benthamiana*, *Danio rerio*, or *Homo sapiens*. The authors therefore proposed that the VdAtuA3–VdNuo1 regulatory axis represents a fungus-specific regulatory mechanism. Importantly, staurosporine bound VdAtuA3, weakened its interaction with VdNuo1, and disrupted mitochondrial homeostasis and virulence. Point-mutation experiments further supported VdAtuA3 as the functional target, while heterologous expression of AtuA homologs from several filamentous fungi restored staurosporine sensitivity, indicating that the target is conserved among these fungi but not in the tested plant and animal systems [59]. The relevance of this example lies not in the conservation of the respiratory machinery itself, but in the possibility of targeting a pathogen-associated regulatory protein associated with conserved mitochondrial functions. Such regulatory interfaces may offer greater selectivity than targeting conserved or structural components.

Other MQC-associated proteins illustrate a progression from biological validation toward chemical tractability. MoGep4, a protein involved in cardiolipin biosynthesis, influences mitochondrial homeostasis and mitophagy in *M. oryzae*. Importantly, inhibition of MoGep4 by alexidine dihydrochloride produced antifungal activity and reduced disease development, providing genetic, mechanistic, and pharmacological evidence for the relevance of this strategy [90]. Moreover, MoCox6 was identified as a mitophagy regulator whose interaction with MoAtg5 and MoAtg14 contributes to mitochondrial quality control. Structural and chemical analyses identified a regulatory interface that could be exploited pharmacologically, and Pan-RAS-IN-1 suppressed fungal growth and pathogenicity [61,62]. These studies suggest that the potential selectivity of mitochondrial targets may lie in pathogen-specific regulatory interactions around conserved mitochondrial proteins.

If disrupting regulation by individual signaling hubs can impair virulence, an even more powerful leverage point may exist further upstream, where the overall set point of MQC activity is established. Master transcriptional and epigenetic regulators illustrate this vulnerability in the control logic. A strikingly parallel phenotype is observed in the necrotroph *T. roseum*, where deletion of the epigenetic reader TrSnt2 increases H3 acetylation, upregulates autophagy-related genes, promotes mitophagy, and simultaneously disrupts ROS homeostasis and membrane integrity, collectively reducing pathogenicity [60]. In this case, the mutant phenotype showed that maintaining autophagic and mitochondrial flux within a narrow range is critical for pathogenesis, and that dysregulation of this balance is as detrimental as deletion.

### Network interconnections as combinatorial vulnerabilities

The interconnected architecture of MQC provides a second potential route for intervention. Mitochondrial quality is maintained through coordinated processes rather than independent pathways: fission facilitates organelle redistribution and can support subsequent mitophagic removal, proteostasis limits the accumulation of damaged proteins, and biogenesis replenishes mitochondrial capacity [13,63]. Perturbing one component might therefore increase the burden on other components and reduce the network’s capacity to compensate.

The interaction between mitochondrial homeostasis and host-derived oxidative stress is particularly relevant. Several fungal mutants affecting mitochondrial functions or MQC-associated processes, including prohibitin-associated mitochondrial homeostasis and mitophagy, display enhanced sensitivity to oxidative stress [49,67,85]. Moreover, host ROS can directly challenge pathogen mitochondria and induce mitophagy; in *Phytophthora sojae*, host-derived H_2_O_2_ promotes a PsPHB2–PsAF5–PsATG8 mitophagy pathway, whereas loss of PsAF5 impairs ROS-induced mitophagy, increases sensitivity to H_2_O_2_, and attenuates virulence [91,92].

The same principle may apply to established fungicides; in *Fusarium graminearum*, mitochondrial fission mediated by FgDnm1 contributes to mitochondrial responses to quinone-outside inhibitors (QoIs) and succinate dehydrogenase inhibitors (SDHIs), and disruption of FgDnm1 alters fungal sensitivity to these compounds [37]. In *Botrytis cinerea*, the mitochondrial transporter BcMtp1 also influences fungicide sensitivity to fluazinam, further supporting the principle that mitochondrial status shapes drug responses [5,55]. These observations support the hypothesis that mitochondrial status can shape fungal responses to established fungicides; however, they do not show that combining an MQC inhibitor with a QoI or SDHI will necessarily produce synergy. Such combinations represent experimental opportunities for resistance management that require quantitative validation in vitro and in vivo.

Beyond interactions with host defenses and existing chemistries, vulnerabilities also arise from the inherent interdependence among MQC processes. Mitochondrial fission, mediated by the Dnm1 complex, is required not only for organelle division but also for subsequent mitophagy, as damaged mitochondria must be fragmented to be efficiently engulfed by autophagosomes [36]. Thus, an inhibitor targeting fission could simultaneously impair both the formation of functional mitochondrial networks and the clearance of dysfunctional organelles, resulting in a compounded defect. The multi-component nature of complexes like the Dnm1 fission machinery (MoDnm1, MoFis1, MoMdv1) and the prohibitin complex (ChPhb1/ChPhb2) provides multiple potential points of intervention, where disrupting protein-protein interactions could affect entire functional modules [36,85]. Targeting such interfaces could theoretically perturb multiple connected functions, but this strategy requires structural information, validated binding sites, and evidence of target engagement. The *T. roseum* TrSnt2 study provides a biological rationale for this possibility because deletion of TrSnt2 altered mitophagy and was accompanied by a disruption of ROS homeostasis, membrane integrity, and pathogenicity.

### Stage-specific intervention points across the infection cycle

The infection cycle of phytopathogenic fungi involves distinct developmental phases with different energetic, metabolic, and stress-management requirements. Available studies indicate that mitochondrial and MQC functions contribute differentially to these phases, creating potential intervention points. For example, foot-cell mitophagy and mitochondrial metabolism influence spore fitness and germination; mitochondrial dynamics contribute to appressorium development and penetration in appressorium-forming pathogens, and mitophagy-associated pathways support mitochondrial fitness during invasive growth and reproduction [13,49]. These stage-specific requirements provide a biological rationale for investigating whether pathogen sensitivity to MQC perturbation can be increased at particular stages.

During pre-penetration development, mitochondrial competence supports conidial viability and germination, whereas mitophagy-associated processes contribute to conidial development in selected pathogens. In *M. oryzae*, MoAtg24- and MoWhi2-associated mitophagy and mitochondrial metabolic processes influence conidiation and germination [13,66]. In *C. camelliae*, CaEch1-mediated mitochondrial quality control functions support conidial germination and appressorium formation [57]. During penetration, mitochondrial fission and broader autophagy-related processes contribute to appressorium development and host invasion, particularly in *M. oryzae* [13,36]. Following penetration, the vulnerability shifts toward mitochondrial remodeling, selective turnover, and stress adaptation. In *M. oryzae*, MoAtg24- and MoAti1-associated mitophagy contributes to invasive growth, while CaSun1-dependent mitophagy supports invasive development in *C. camelliae* [13,51,57]. In other pathogens, mitochondrial competence and homeostasis similarly become important for sustained colonization [53,55,88]. These observations suggest that perturbations of mitophagy or mitochondrial dynamics may have particularly strong biological effects during invasive growth, although the precise stage of maximal sensitivity will vary among pathogens. The connection between mitochondrial fission and mycotoxin production in *F. graminearum* further highlights how MQC influences not only pathogen fitness but also virulence and food safety outcomes [37]. During reproduction and secondary spread, mitochondrial quality becomes relevant again to produce viable spores. Mitophagy-associated processes in *M. oryzae* and other filamentous fungi influence conidiation and developmental fitness [37,56,65,66]. This provides a biological basis for investigating whether MQC perturbation can reduce inoculum production or spore quality, thereby interrupting disease cycles.

### From MQC target validation to antifungal development

Translating MQC vulnerabilities into antifungal strategies requires a clear distinction between genetic evidence that a mitochondrial process contributes to pathogenicity and pharmacological evidence that the corresponding process can be selectively targeted. Several studies have begun to bridge this gap, ranging from compounds targeting pathogen-specific mitochondrial regulators to inhibitors of mitochondrial respiratory functions that indirectly perturb mitochondrial homeostasis. However, these approaches differ substantially in target specificity and the strength of their validation. Table 2 therefore summarizes pharmacological interventions reported in phytopathogenic fungi. For each approach, the table considers the proposed target or process, and the available evidence for antifungal or disease-control activity.

**Table 2.** Pharmacological approaches targeting mitochondrial quality control and mitochondrial vulnerabilities in phytopathogenic fungi.

Fungi	Compound	Target	MQC relationship	Antifungal / disease-control evidence	References
*M. oryzae*	Alexidinedihydrochloride (AXD)	MoGep4; cardiolipin biosynthesis	Direct MQC-associated; MoGep4 regulates mitophagy	Inhibited growth of 10 plant pathogens; reduced rice blast and Fusarium head blight; field efficacy reported	[90]
*M. oryzae*	Pan-RAS-IN-1	MoCox6 regulatory interface	Direct MQC; inhibits MoCox6-associated mitophagy	Inhibited fungal growth, appressorium formation and infection; suppressed rice blast; broad-spectrum antifungal activity	[61,62]
*V. dahliae*	Staurosporine	VdAtuA3–VdNuo1 regulatory axis	MQC-associated / mitochondrial homeostasis rather than core MQC	EC_50_ = 1.60 μM; reduced Verticillium wilt on cotton; activity against several filamentous fungi	[59]
*M. oryzae*	Mdivi-1	Proposed Dnm1-dependent mitochondrial fission	Direct MQC process implicated, but compound specificity unresolved	Reduced pathogenicity of *M. oryzae*; no definitive target-engagement evidence	[13,36]

Recent studies illustrate this progression. MoGep4 provides an example in which genetic evidence was followed by chemical inhibition and disease-control experiments using alexidine dihydrochloride [90]. MoCox6 provides a more advanced example, combining mechanistic mitophagy analysis with structural characterization, chemical screening, and identification of Pan-RAS-IN-1 as a compound that perturbs the MoCox6-associated regulatory system. The VdAtuA3 study further illustrates how a pathogen-associated mitochondrial regulator can provide a pharmacological entry point into the mitochondrial machinery [59]. These studies show that MQC-associated vulnerabilities are moving beyond genetic observations toward experimentally validated chemical interventions.

Candidate compounds should nevertheless be separated into at least four categories: direct MQC-targeting compounds, compounds that modulate specific MQC regulators, mitochondrial toxicants, and general respiratory inhibitors. A compound that causes mitochondrial depolarization or inhibits respiration might strongly affect fungal growth without engaging a particular MQC target. Conversely, a compound that alters mitophagy without immediately collapsing mitochondrial respiration might provide more informative evidence of pathway-specific target engagement. Mdivi-1 illustrates this distinction: its ability to phenocopy Dnm1-associated phenotypes supports the biological importance of mitochondrial fission, but it cannot be regarded as definitive evidence of selective Dnm1 target engagement [93].

## Conclusion

Mitochondrial quality control provides an important mechanistic link between mitochondrial integrity and fungal pathogenicity. Across phytopathogenic fungi, mitochondrial dynamics, biogenesis, proteostasis, and mitophagy help maintain the mitochondrial competence required for development, stress adaptation, host penetration, colonization, and reproduction. However, evidence strength differs among pathogens and MQC layers. Direct studies of mitophagy and mitochondrial dynamics provide the clearest evidence for MQC-dependent pathogenicity. In contrast, studies of mitochondrial metabolism, respiration, transport, and oxidative homeostasis provide complementary evidence for the functional capacity that MQC must preserve.

The evidence assembled here supports three complementary dimensions for investigating MQC’s translational potential. First, pathogen-specific regulatory proteins, adaptors, and protein–protein interaction interfaces may provide opportunities for selective intervention within otherwise conserved mitochondrial systems. Second, the interconnected architecture of MQC creates potential combinatorial vulnerabilities because perturbation of one quality-control process may increase dependence on other components or reduce tolerance to mitochondrial and host-derived stresses. These interactions provide experimentally testable opportunities for combination strategies. Third, MQC requirements vary across infection stages and pathogenic lifestyles. Stage-specific biological dependence therefore supports investigating temporally optimized interventions, but experimentally demonstrated therapeutic windows require conditional or time-resolved perturbation and appropriate pharmacological validation.

However, several challenges must be addressed before MQC can be translated into practical antifungal development. Genetic disruption establishes biological importance but does not establish chemical tractability or target engagement. Future studies should therefore combine genetic validation with biochemical assays, structural analysis, resistance-based target confirmation, quantitative pharmacology, and *in planta* efficacy testing. Fungal–host selectivity will be particularly important because many core MQC components are evolutionarily conserved. Evaluation should also consider effects on beneficial fungi, tissue penetration, formulation, compound stability, environmental persistence, cost, and the potential for resistance evolution. Emerging technologies may accelerate this process. High-throughput screening can identify compounds that perturb pathogen-specific MQC regulators or regulatory interfaces. At the same time, structural biology and AI-assisted protein and protein-complex modeling can help identify fungal-specific pockets and interaction surfaces for structure-guided compound discovery. Such computational approaches remain hypothetical until supported by biochemical target engagement and genetic evidence. Combination strategies with established fungicides or host-defense activation likewise represent testable avenues for resistance management. Overall, current evidence supports MQC as an important component of fungal pathogenicity and provides a framework for identifying pathogen- and stage-dependent mitochondrial vulnerabilities. The major opportunity is therefore not simply to inhibit mitochondria broadly, but to determine whether specific MQC mechanisms or regulatory interfaces can be perturbed selectively enough to compromise fungal infection while minimizing effects on the host and beneficial microorganisms. Establishing this progression from MQC mechanism to mitochondrial functional consequence, infection-stage requirement, target engagement, and disease-control efficacy will determine whether MQC can ultimately become a practical component of sustainable plant disease management.

## Data Availability

This review is based exclusively on previously published studies. No new datasets were generated or analyzed. All sources used in this review are cited in the reference list.
